# Supplementary material for: Metabolome-Wide, Phylogenetically Controlled Comparison Indicates Higher Phenolic Diversity in Tropical Tree Species
Source: Plants (Basel). 2021 Mar 16;10(3):554. doi: 10.3390/plants10030554 (PMC7998528; doi:10.3390/plants10030554)

Supplementary Materials 'S1'

Extended Methods

Analyses of the Metabolomic Profiles

The leaves were processed following [46]. Briefly, the leaves were frozen in liquid nitrogen immediately after sampling. The samples were ground with a ball mill at 1500 rpm for 3 min and then extracted using a 1:1 methanol:water solution. The extracts were stored at -80 °C until the analysis of metabolites. Each extract was analyzed twice using an UltiMate 3000 high-performance liquid chromatograph (HPLC) coupled to an LTQ Orbitrap XL high-resolution mass spectrometer (HRMS) (Thermo Fisher Scientific, Waltham, USA), first in the positive-ion mode and then in the negative-ion mode. The HPLC-HRMS system (controlled by Xcalibur 2.2; Thermo Fisher Scientific, USA) was run in gradient mode. Solvents A and B were acetonitrile and 0.1% acetic acid, respectively. Both A and B mobile phases were filtered and degassed for 10 min in an ultrasonic bath prior to use. Gradient-elution chromatography was performed starting with 10% A and 90% B and maintained for 5 min. The percentage of A was increased to 90% within 5–20 min. This composition was then maintained for 5 min, after which the system was equilibrated to initial conditions (10% A and 90% B) over 5 min. Hypersil Gold reverse-phase column (150 × 2.1 mm and the internal diameter of 3 μm; Thermo Fisher Scientific) was used to separate metabolites. The column temperature and flow rate were set at 30 °C and 0.3 mL min^{-1}, respectively.

The HRMS was equipped with a HESI II (heated electrospray ionization) source and was operated in full-scan mode with a resolution of 60 000. Full-scan spectra were acquired for the mass range m/z 50–1000 in the positive mode and 65–1000 in the negative mode. The resolution and sensitivity of the HRMS were controlled by the injection of a mixed standard after every 30 samples. The resolution was also checked with the aid of lock masses (phthalates). Samples were re-analyzed (MS/MS) under the same conditions, but with selecting the top three parental ions in each scan. Parental ions with a minimum intensity of 500 area were fragmented by CID (normalized collision energy of 35, activation Q of 0.25, and activation time of 90 ms) and analyzed in an ion trap mass spectrometer (ITMS™). Recordings from both the high-resolution mass spectrometer and the diode array detector (DAD) monitored at wavelengths of 254, 272, 274, and 331 nm were saved for further verification and evaluation of the results.

Chromatogram Alignment and Metabolite Quantification

The raw data from the HPLC-HRMS system were processed and compared using XCMS [30]. XCMS scripts (https://xcmsonline.scripps.edu/) were used for the nonlinear alignment of the data in the time domain and the automatic integration and extraction of the peak intensities. The peak detection algorithm was based on cutting the HPLC-HRMS data into slices a fraction of a mass unit (0.1 m/z) wide and then operating on the individual slices in the chromatographic time domain. Peaks were identified in the individual samples and then matched across samples to calculate the deviations of retention time and to compare relative ion intensities. MS1 spectra were filtered, establishing a noise threshold at 2E04 and a minimum peak height at 2.5 E05. Peaks were smoothed and deconvoluted using a local minimum search algorithm (95% chromatographic threshold, minimum retention range of 0.2 min, the minimum relative height of 5%, and minimum ratio top/edge of 0.5). Chromatograms were aligned using the algorithm with a tolerance of 5 ppm of m/z and 0.2 min of retention time. Normalized peak areas were used for quantification, and their values were log-transformed before statistical analysis. MS/MS data were only used for metabolite identification.

The molecular formula was assigned to peaks with a signal-to-noise ratio greater than four, according to stringent criteria with elemental combinations of $C_{1-100}H_{1-250}O_{1-100}N_{0-4}P_{0-1}S_{0-1}$ [32,47]. Besides, molecular formula with extreme heteroatom (with N = 4 and S = 1) assignments, no systematic trends in P-containing compounds, S-containing

compounds, or N-containing compounds with N > 2 were excluded from the data. A threshold was applied to compare such a diverse tree sample set, the dataset detection limit (S/N > 4), where all peaks with a sum-normalized intensity lower than the smallest peak with this detection limit in the sample are not considered. This peak processing method resulted in a dataset of 2461 peaks, having formula assignments. Molecules were assigned to compound groups based on the stoichiometry of their molecular formula. The groups relevant to our study are delineated by the aromaticity index (AI) [32,47] and H/C cutoffs [4]: combustion-derived polycyclic aromatics (AI > 0.66), vascular plant-derived polyphenols (0.66 ≥ AI > 0.50), highly unsaturated and phenolic compounds (AI ≤ 0.50 and H/C < 1.5), and aliphatic compounds (2.0≥ H/C ≥1.5).

Data Analysis

The metabolites were quantified after eliminating peaks that were not consistently representative (mass/RT in at least three samples of any variant; each sample was split and randomly queued into two batches of LC-MS runs). The peak areas corresponding to each metabolite were normalized based on total peak areas in the sample. Neutral masses obtained in positive and negative modes were evaluated to avoid duplicates (same RT and neutral mass in the different modes), retaining the most intense peaks. We then calculated metabolite richness (i.e. number of different compounds) and chemical diversity (CD) for each sample as:

$$\mathrm{CD} = -\sum_{i=0}^{n} pi \times \ln pi$$

where p_i is the peak area of a compound relative to the total peak area, and the sum includes all compounds detected (after [25].

We used the phylogenetic tree from [31] and updated it by [36] to introduce the evolutionary relationships between the species to our models. The Bayesian multilevel models were built using the R brms package [34]. The richness and chemical diversity of the entire metabolome and for each family of metabolites were generally modeled with the region of origin (temperate vs tropical) as a two-level fixed-effects categorical factor and the phylogenetic covariance matrix and species identity as random factors, thus also accounting for evolutionary relationships and intraspecific variance [27,34,35]. Before following a normal distribution with mean = 0 and sd = 10 times the standard deviation of the response variable was used to sample the coefficient and a half Student-t prior with 3 degrees of freedom for the random factors. We ran the models with four chains of 2000 iterations, burning-in the first 1000. All models converged with a potential scale reduction statistic $\hat{R}$<1.01. Hypothesis testing of the fixed effects parameters was carried out by checking 95% credible intervals and computing their associated evidence ratios. An evidence ratio of X can be interpreted as that the alternative hypothesis (e.g. that tropical species have a greater diversity of phenolic compounds) being X times more probable than the null hypothesis. This was calculated using the *hypothesis* function from the R brms package [34].

Supplementary Figure S1

Figure 1. Phylogenetic relationships of the species [31,32].

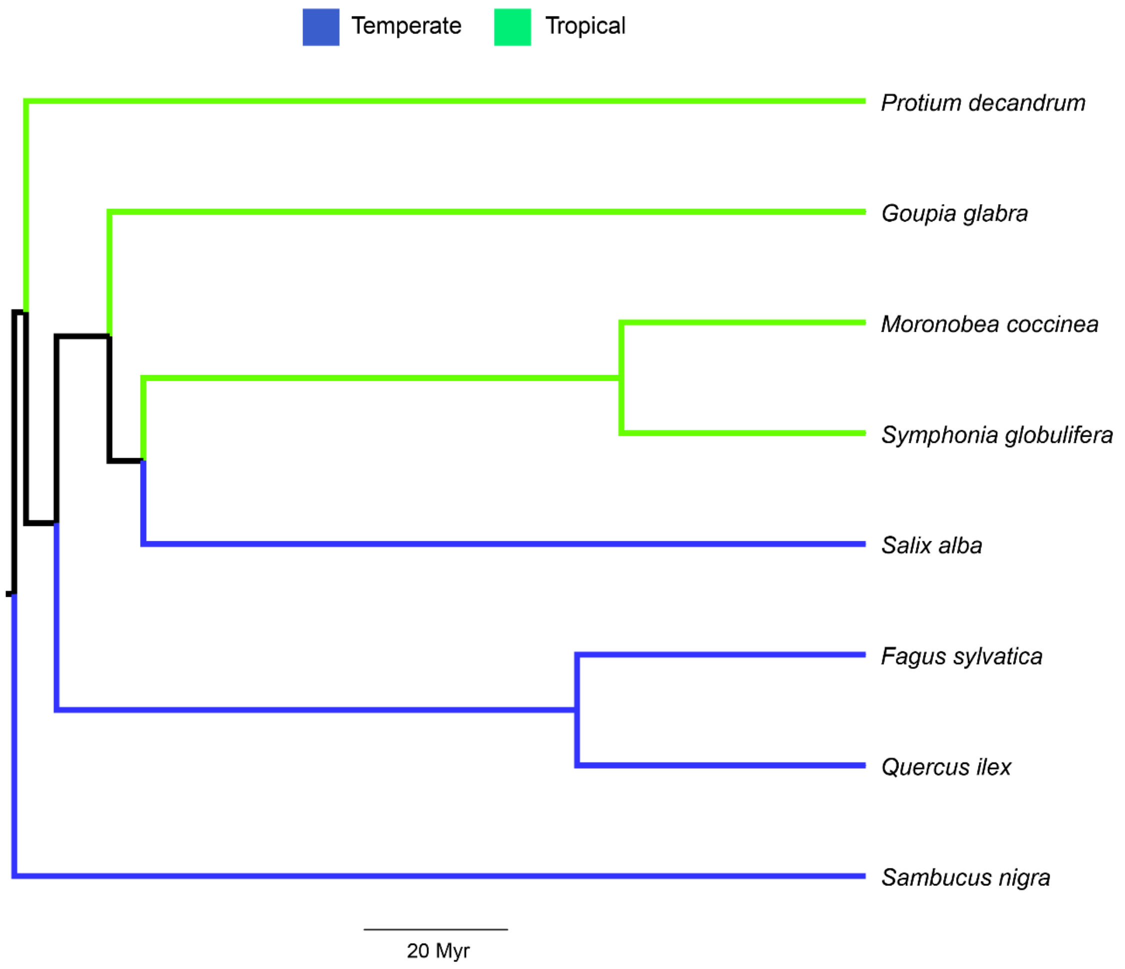

Supplement: Supplementary file 1 [file plants-10-00554-s001.pdf]
